# Versatile Genome Engineering Techniques Advance Human Ocular Disease Researches in Zebrafish

**DOI:** 10.3389/fcell.2018.00075

**Published:** 2018-07-12

**Authors:** Si-Si Zheng, Ru-Yi Han, Lue Xiang, You-Yuan Zhuang, Zi-Bing Jin

**Affiliations:** ^1^Division of Ophthalmic Genetics, Laboratory for Stem Cell and Retinal Regeneration, Institute of Stem Cell Research, The Eye Hospital, Wenzhou Medical University, Wenzhou, China; ^2^National International Joint Research Center for Regenerative Medicine and Neurogenetics, Wenzhou Medical University, Wenzhou, China; ^3^State Key Laboratory of Ophthalmology, Optometry and Visual Science, Wenzhou, China

**Keywords:** CRISPR/Cas9, genome editing, ocular development, zebrafish, morpholino

## Abstract

Over recent decades, zebrafish has been established as a sophisticated vertebrate model for studying human ocular diseases due to its high fecundity, short generation time and genetic tractability. With the invention of morpholino (MO) technology, it became possible to study the genetic basis and relevant genes of ocular diseases *in vivo*. Many genes have been shown to be related to ocular diseases. However, the issue of specificity is the major concern in defining gene functions with MO technology. The emergence of the first- and second-generation genetic modification tools zinc-finger nucleases (ZFNs) and TAL effector nucleases (TALENs), respectively, eliminated the potential phenotypic risk induced by MOs. Nevertheless, the efficiency of these nucleases remained relatively low until the third technique, the clustered regularly interspersed short palindromic repeats (CRISPR)/CRISPR-associated protein 9 (Cas9) system, was discovered. This review highlights the application of multiple genome engineering techniques, especially the CRISPR/Cas9 system, in the study of human ocular diseases in zebrafish.

## Introduction

Zebrafish, a fresh water teleost fish, has been used in genetic studies due to advantages in genetic modification, reproduction and developmental duration (Patton and Zon, 2001). This tiny fish displays three distinct eye traits. First, the eyeball of zebrafish larva is very large relative to its overall size (Goldsmith, 2001). Second, the eye develops quite rapidly after fertilization in water. It can respond to light by 72 h postfertilization (hpf), as the retina at this time point already resembles that in the adult stage (Lieschke and Currie, 2007). Third, the ocular structure of this fish is anatomically similar to that of humans (Link and Collery, 2015). The zebrafish optic lumina forms by 14 hpf, and then, the optic vesicle undergoes a similar series of morphogenetic movements between 16 and 20 hpf as in humans (Richardson et al., 2017). Compared to the human retina, the mature zebrafish retina is composed of three nuclear layers, two plexiform layers and all of the main retinal cell types, including photoreceptor cells, horizontal cells, bipolar cells, amacrine cells, Müller glial cells and ganglion cells (Richardson et al., 2017). In addition, 70% of human genes have at least one ortholog in zebrafish, making zebrafish very genetically tractable (Richardson et al., 2017). The aforementioned traits enable a widespread investigation of human ocular diseases in this vertebrate model.

**Figure 1:**
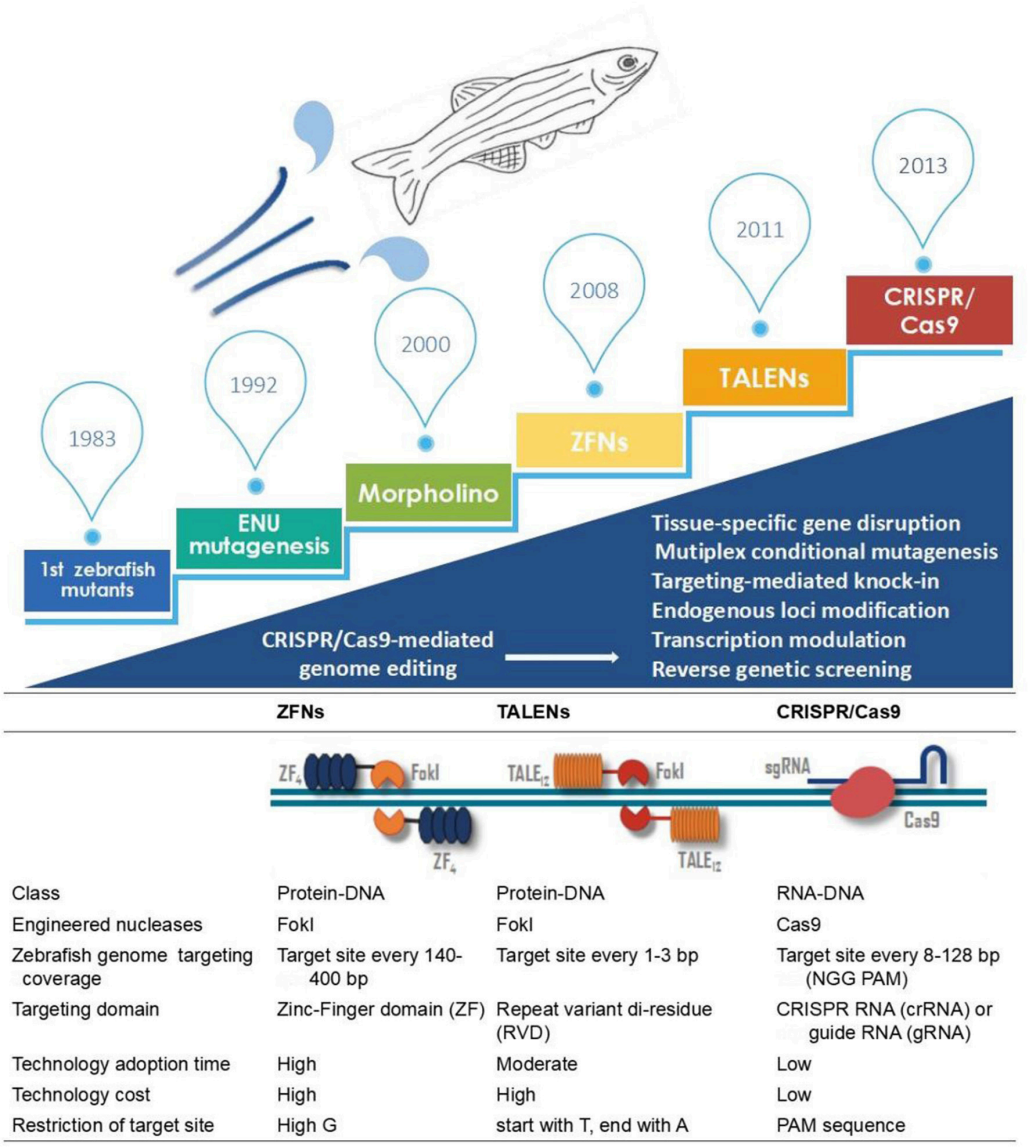
Outline of the developing utilities in zebrafish disease modeling. Key technological developments in zebrafish research are shown in the above timeline. Comparisons of ZFNs, TALENs and the CRISPR/Cas9 system are described below.

Diverse genome editing technologies, such as zinc-finger nucleases (ZFNs), TAL effector nucleases (TALENs) and the clustered regularly interspersed short palindromic repeats (CRISPR)/CRISPR-associated protein 9 (Cas9) system, have emerged to address the demand for genomic modification in zebrafish (Woong et al., 2013). These genome editing technologies are based on engineered endonucleases that enable the induction of targeted DNA double-strand breaks (DSBs) at specific sites. Once DNA DSBs occur, the cleaved DNA is repaired by nonhomologous end joining (NHEJ) or homology-directed repair (HDR). Because of the high DNA cutting efficiency and flexibility of CRISPR/Cas9 compared to other reverse genetic approaches, as described in Figure 1, the CRISPR/Cas9 approach is more widely used to modify endogenous genes in a wide range of cell lines and living organisms. This review will focus on the most frequently used genetic modification tools, including morpholinos (MOs), ZFNs, TALENs and CRISPR/Cas9, and their roles in studying human ocular diseases in zebrafish.

## MO-mediated knockdown approach in studying ocular diseases in zebrafish

In 1977, Summerton and colleagues developed a powerful reverse genetic approach using MOs to block the translation process of particular mRNA or to interfere with the mRNA splicing process (Summerton and Weller, 1997; Summerton, 1999). Utilizing this convenient reverse genetic technique, embryologists and ophthalmologists have isolated many genes that are related to ocular diseases over recent decades. For example, Joubert syndrome (JBTS) is a type of human diseases associated with numerous ciliopathic defects, and patients display retinopathy, ataxia and cognitive impairment (Sayer et al., 1999; Valente et al., 1999). By combining the MO knockdown technique with the fast-developing zebrafish, researchers have identified a dozen genes associated with JBTS. JBTS-causing genes, including AHI (Elsayed et al., 2015), CC2D2A (Bachmann-Gagescu et al., 2011, 2015), CEP290 (Baye et al., 2011), CSPP1 (Tuz et al., 2014), INPP5E (Luo et al., 2012; Xu et al., 2017), PDE6D (Thomas S. et al., 2014), and POC1B (Roosing et al., 2014; Zhang et al., 2015), have been verified in zebrafish models. Specifically, knocking down inpp5e by MOs lead to microphthalmia, pronephros cysts and pericardial effusion in zebrafish. A mechanistic study revealed that this gene functions as a key regulator of cell polarity by interacting with PtdIns(3,4,5)P3, PtdIns(4,5)P2, and Ezrin (Luo et al., 2012; Xu et al., 2017). This molecular process is critical for cilia formation and thus affects ocular development. In addition, controlling the expression of another cilia-related gene, poc1b, with MO knockdown resulted in similar photoreceptor sensory cilium defects as those observed in the inpp5e morphant (Roosing et al., 2014; Zhang et al., 2015). The MO knockdown technique has underpinned the initial growth of gene discovery, which was valuable for human ocular disease studies in zebrafish.

With the expansion of this reverse genetic approach, researchers began to worry about the specificity of this technique. Although not all types of methods were covered, the following two types of treatments could reduce the potential off-target effects in zebrafish MO studies (Nasevicius and Ekker, 2000). One method is the coinjection of several MO oligos to lower or eliminate dose-dependent off-target RNA interactions. The other method is performing mRNA rescue experiments. However, in 2014, Law and Sargent claimed that the pak4 MO-induced morphant could not be recapitulated in the mutant generated by TALENs, which was also corroborated by robust RNA rescue experiments (Law and Sargent, 2014). In the next year, Kok FO et al. observed that among the twenty specific mutants, only a small proportion of candidate genes recapitulated published MO-induced morphants (Kok et al., 2015). Similarly, Shmukler et al. described that unlike the MO-induced morphant, the piezo1 knockout model was not associated with anemia in zebrafish (Shmukler et al., 2015). Again, researchers found inconsistent phenotypic consequences between two zebrafish models generated by MOs and TALENs when studying klf2a, egfl7, tmem88a, and atoh8 genes (Novodvorsky et al., 2015; Rossi et al., 2015; Eve et al., 2017; Place and Smith, 2017). There are many explanations for these discrepancies, including knockdown reagent toxicity, off-target effects and transcriptional adaptation and/or genetic compensation in mutants (Rossi et al., 2015; El-Brolosy and Stainier, 2017; Joris et al., 2017). Regarding the poor correlation between some mutants and morphants, confirmation of the observed phenotypes by the use of a nuclease-induced mutant strain may be a more reliable way to affirm the morphant phenotypes.

## Utilizing the first two genomic modification tools, ZFNs and TALENs, to survey ocular diseases in zebrafish

As reverse genetic approaches have become popular, particularly programmable site-specific nucleases, more studies have utilized these approaches in zebrafish. ZFNs are the first engineered nucleases that were broadly used for DNA manipulation (Bibikova et al., 2003). Two groups took the lead in making ZFNs that could introduce heritable mutations into the zebrafish genome (Doyon et al., 2008; Meng et al., 2008). Both groups disrupted “proof-of-principle” loci with ZFN technology. Doyon et al. injected gol-targeted ZFN-encoding mRNA into one-cell embryos and then detected somatically induced mutations. Although golb1 heterozygotes exhibit normal dark eye pigmentation as normal, the authors observed clones of unpigmented cells in the eye, as expected. Subsequently, another group created clrn1 mutants using ZFNs (Gopal et al., 2015). Mutation in the clrn1 gene caused Usher syndrome type III, which is characterized by the progressive loss of vision and hearing in patients. These findings suggested that ZFNs could be employed to induce gene expression in zebrafish for further mechanistic studies of eye phenotypes.

However, although ZFNs have been optimized over decades (Miller et al., 2007; Foley et al., 2009), this method still lacks flexibility and efficiency in generating mutants. An alternative, efficient method for direct genomic manipulation was thus required.

TALENs, the newly developed engineered nuclease, can be more easily engineered than ZFNs (Bedell et al., 2012) (Figure 1; Table 1). Using this new technology, researchers have found strong evidence of a link between ocular diseases and target genes, such as MAB21L2, EFTUD2, PITX2, AHI1 and B3GLCT (Deml et al., 2015a,b; Lessieur et al., 2017; Weh et al., 2017; Hendee et al., 2018). The capability of TALENs to quickly and efficiently alter genes helped extend and confirm the results obtained in zebrafish using MOs. For example, lens abnormalities observed in the TALEN-based αA-crystallin knockout zebrafish line provided strong evidence of the conservation and importance of α-crystallin in lens development (Zou et al., 2015). Lewis TR et al. used TALENs to generate an independent TALEN-mediated allele, osm-3/kif17^mw405^. The new line of zebrafish successfully displayed abnormal ocular phenotypes, such as outer segment developmental delay in both size and density, but failed to validate OS morphogenesis in osm-3/kif17 morphants (Lewis et al., 2017). However, both ZFNs and TALENs have shortcomings, including a low transmission efficiency, long technology adoption time and high cost (Figure 1).

**Table 1:** Optimization of the CRISPR/Cas9 system.

**Cas9 orthologs**	**Advantages**	**Application in zebrafish**	**References**
SpCas9 VQR	Altered PAM specificity (NGAN and NGCG PAMs)	Broaden the targeting range of SpCas9 in zebrafish embryos	Kleinstiver et al., 2015
SpCas9 EQR	Altered PAM specificity (NGAG PAMs)		Kleinstiver et al., 2015
SpCas9 VRER	Altered PAM specificity (NGCG PAM)		Kleinstiver et al., 2015
zCas9	Zebrafish codon-optimized Cas9 protein	A CRISPR/Cas9-meditated intron-targeting knock-in strategy	Li et al., 2015
SaCas9	Smaller-size protein (1,053 amino acids, 3.16 kbp)		Kleinstiver et al., 2015
CjCas9	Smaller-size protein (984 amino acids, 2.95 kbp)		Kim et al., 2017
Cas9n	Produce single-strand nicks at target sites	A programmable, efficient single-base editing system	Zhang et al., 2017
Cpf1	Produce sticky ends at target sites	Increase homology-directed repair and mutagenesis efficiency in the zebrafish genome	Moreno-Mateos et al., 2017
xCas9	Altered PAM specificity (NG,GAA and GAT PAMs)		Hu et al., 2018

*VQR, D1135V/R1335Q/T1337R; EQR, D1135E/R1335Q/T1337R; VRER, D1135V/G1218R/R1335E/T1337R*.

## Efficient CRISPR/Cas9-mediated gene editing in zebrafish models of human ocular diseases

The efficiency and specificity of the CRISPR/Cas9 system compared to traditional chemical-induced mutagenesis techniques makes it the preferred method. Here, we discuss the most recent achievements in human ocular disease modeling with the CRISPR/Cas9 system in zebrafish.

Leber congenital amaurosis (LCA) is a leading cause of early-onset blindness in children, and CCT2 is one of the eight encoded proteins of chaperonin containing T-complex protein-1 (CCT). In a recent publication, Minegishi Y et al. introduced a tiny in-frame deletion mutation in the cct2 gene with CRISPR/Cas9 (Minegishi et al., 2018). CCT2-L394H-7del mutants displayed small eyes with reduced pigmentation and could be specifically rescued by CCT2 RNA injection. This genotype-phenotype consistency and phenotypic details could not be achieved in previous works by chemical mutagenesis due to its uncontrollability (Golling et al., 2002).

Microphthalmia is a type of eye malformation with a total axial length at least two standard deviations below that of the population age-adjusted mean (<21 mm in the adult) (Weiss et al., 1989; Verma and Fitzpatrick, 2007). More than 20 genes have been implicated in microphthalmia, including SOX2 and SMCHD1 (Chassaing et al., 2016; Shaw et al., 2017; Huang et al., 2018). Among these genes, SOX2 is the most frequent culprit gene accounting for a minority (10–15%) of the genetic drivers. Recently, Nicolas Chassaing et al. reported significant enrichment of PTCH1 variants, thereby extending the SOX2 regulatory network (Chassaing et al., 2016). Their CRISPR/Cas9-mediated knockout zebrafish successfully displayed reduced eye size resembling that in patients, while the first-generation morphants failed to display this phenotype.

The CRISPR/Cas9 system can not only help reveal missing phenotypes in MO-mediated experiments but also eliminate the irrelevant phenotypes caused by potential off-target effects. B-crystallin, a small heat shock protein, was considered to be associated with severe muscle dystrophy in previous work using MO-mediated knockdown zebrafish (Bührdel et al., 2015). However, CRISPR/Cas9-mediated B-crystallin knockout zebrafish exhibited lens abnormalities and hypersusceptibility to pericardial edema under stress but not severe muscle phenotypes (Mishra et al., 2018).

Apart from isolated microphthalmia, Bosma arhinia microphthalmia syndrome (BAM) is a rare developmental malformation lead to an absent nose and microphthalmia. As there were multiple relevant phenotypes, the off-target effects had to be considered. Therefore, Natalie D Shaw et al. used CRISPR/Cas9-edited smchd1 knockout zebrafish as well as knockout mice to verify the MO knockdown results (Shaw et al., 2017). The knockout zebrafish successfully recapitulated the phenotypes displayed by the morphants, including defects of the ethmoid plate, terminal nerve and eye size. In other words, CRISPR/Cas9 systems provide double assurance in function identification. Interestingly, knockout mice in this study failed to display a craniofacial phenotype, raising another issue about genetic background in disease modeling.

Due to different genetic backgrounds, it is quite common to find inconsistent phenotypes despite manipulation of the same gene between species. For example, mutations in membrane frizzled-related protein (MFRP) have been found to be associated with microphthalmia, but previous studies on mfrp mutant mice did not show this phenotype. However, when Collery RF et al introduced stop codons in the zebrafish mfrp gene with the CRISPR/Cas9 system, the knockout zebrafish successfully mimicked patient phenotypes (Collery et al., 2016). The simplicity and convenience of the CRISPR/Cas9 system cannot be achieved by ZFNs nor TALENs due to their tedious construction system (Schierling et al., 2012; Gaj et al., 2013; Xiang et al., 2017). With the CRISPR/Cas9 system, researchers can easily achieve single or multiple gene manipulation regardless of species, thus solving the problem of different genetic backgrounds.

## Expansion of the application of the CRISPR/Cas9 system in zebrafish studies

In addition to the simple knockout strategy with the CRISPR/Cas9 system, there are further uses of this technology, such as the conditional knockout strategy and knock-in strategy. First, a conditional knockout strategy is developed via various methods, such as optimization of the CRISPR-based vector system with a tissue-specific promoter driving Cas9 expression (Ablain et al., 2015) and combining the CRISPR/Cas9 system with the Gal4/UAS binary system (Di Donato et al., 2016) or Cre/loxP system (Liu et al., 2017; Kesavan et al., 2018). Gokul Kesavan et al. generated zebrafish lines by inserting a codon-optimized CreERT2 transgene at the otx2 gene locus, and this manipulation led to efficient recombination (Kesavan et al., 2018).

Second, the knock-in strategy with the CRISPR/Cas9 system also works smoothly. For example, with homology-independent DNA repair, Thomas O. Auer et al. showed the high efficiency of CRISPR/Cas9-mediated knock-in of >5.7-kb-long DNA cassettes into zebrafish (Auer et al., 2014). Moreover, in 2015, Li et al. used the CRISPR/Cas9 system to introduce intron targeting-mediated *EGFP* knock-in at the zebrafish tyrosine hydroxylase locus (Li et al., 2015).

Last, using the CRISPR/Cas9 system combined with a blue-light-activated EL222 system (the modified EL222 system), researchers designed an optogenetic gene expression system optimized for a large range of induction and fine spatial precision with low toxicity in zebrafish (Reade et al., 2017). Furthermore, Yukiko Kimura et al. generated transgenic zebrafish that had cell-type-specific Gal4 or reporter gene expression (Kimura et al., 2014). It is therefore possible that there are additional potential applications of the CRISPR/Cas9 system worth investigating.

## Optimization of the CRISPR-Cas9 system in zebrafish genome editing

In addition to successful mutant modeling, seeking more reliable methods to identify CRISPR/Cas9-induced mutants and detect mutation frequency is crucial for further study of gene function. The traditional methods include polymerase chain reaction (PCR)/restriction enzyme (RE) assay, T7 endonuclease I (T7EI) assay, Surveyor nuclease assay, PAGE-based genotyping assay, and high-resolution melting (HRM) analysis-based assay (Thomas H. R. et al., 2014; Zhu et al., 2014). In 2017, Yufeng Hua et al. developed a new, efficient method called annealing at critical temperature PCR (ACT-PCR), which enabled novel mutant identification and efficient confirmation of CRISPR/Cas9-mediated gene editing in zebrafish (Hua et al., 2017).

However, the following two issues cannot be avoided with the sophisticated CRISPR/Cas9 system: genome editing efficiency and off-target rates. In recent years, researchers have developed two main strategies, including modifying the Cas9 protein and finding Cas9 orthologs to tackle these issues.

First, Cas9n was generated as an alternative to the Cas9 RNA-guided nuclease. This nickase, with one sgRNA, is capable of introducing single-strand nick rather than DSBs (Table 1). Cas9n, with two different sgRNAs, can mediate highly specific genome editing and reduce potential off-target mutagenesis by wild-type Cas9 (Jinek et al., 2012; Trevino and Zhang, 2014). Using cytidine deaminase fused to Cas9 nickase, Zhang et al. revealed a programmable, highly efficient single-base editing system in zebrafish, remarkably increasing the precision of genome editing (Zhang et al., 2017).

Second, three smaller-size Cas9 orthologs, Streptococcus thermophilus Cas9 (St1Cas9), Staphylococcus aureus Cas9 (SaCas9) and Campylobacter jejuni (CjCas9), were also shown to be efficient (Kleinstiver et al., 2015; Kim et al., 2017) (Table 1). The results suggested that Cas9s from other species could improve protospacer adjacent motif (PAM) specificity, thereby broadening the use of the CRISPR system (Kleinstiver et al., 2015). Subsequently, Zhang et al. found another single RNA-guided endonuclease, Cpf1 protein, that could produce sticky ends at the target site (Zetsche et al., 2015) (Table 1). The gene targeting rates using Cpf1 in mice can reach or even exceed Cas9-targeting rates. With further knowledge of Cpf1, the authors showed that LbCpf1 activity combined with optimized single-stranded DNA (ssDNA) donors could markedly increase HDR and efficiently mutagenize the genomes of zebrafish (Moreno-Mateos et al., 2017). To further eliminate the restriction of PAM, Hu et al. used phage-assisted continuous evolution (PACE) to develop an SpCas9 variant (xCas9) with a wide range of PAM sequences, including NG, GAA, and GAT, that could significantly improve current approaches for genome editing. However, the mechanism of xCas9 is poorly understood, which indicates that the application of xCas9 in zebrafish genome engineering still requires exploration (Hu et al., 2018).

In addition, there are several other approaches to increase the efficiency of genome editing and reduce off-target rates, such as improved design tools for single guide RNA sequences (Moreno-Mateos et al., 2015; Prykhozhij et al., 2015; Haeussler et al., 2016) and high-throughput functional genomics workflows (Varshney et al., 2016). Additionally, Kelly A. Smith et al. found that single nucleotide polymorphisms (SNPs) within the target site insulate genome editing. This feature can be further exploited to increase the efficiency of cis genome editing in the zebrafish model (Capon et al., 2017). In addition, Xie et al. reported a method based on prior microinjection of zebrafish oocytes and *in vitro* fertilization (IVF) to improve the efficiency of genome editing and germline transmission in zebrafish (Xie et al., 2016).

## Perspective

Zebrafish provide notable advantages in studying ocular diseases due to their large eye-body ratio, genetic tractability, external fertilization, and rapid development of the visual system. Strikingly, zebrafish have two tremendous advantages over other animal models. One is that zebrafish have a characteristic cone-dominated retina, which is more similar to humans than mouse (Bilotta et al., 2001). The zebrafish retina also possesses a strong ability to regenerate lost neurons via Müller glia cell regeneration after injury (Meyers et al., 2012). Further studies utilizing these two features in zebrafish can provide a novel perspective of genetic diagnosis and mechanistic studies in human ocular-related diseases and may provide unanticipated opportunities for therapy. Despite the limitations of applying a smaller CRISPR/Cas9 system in zebrafish-based disease treatment, this method can help us both understand the disease mechanism and explore the possibility of regenerating neurons in ocular tissues (Ge et al., 2014; Zhang et al., 2016; Leung et al., 2018). Overall, with the new CRISPR/Cas9 system, researchers have versatile genetic modification tools to both edit genes and regulate their expression networks with ease. Without doubt, the application of CRISPR-Cas9 gene editing in zebrafish can play a robust role in human ocular disease therapy.

## Author contributions

S-SZ, R-YH, and LX contributed equally to this review. S-SZ wrote utilizing the first two genomic modification tools, ZFNs and TALENs, to survey ocular diseases in zebrafish and the CRISPR-Cas9 system in zebrafish genome editing and its optimizations sections. R-YH wrote CRISPR/Cas9-mediated gene editing in zebrafish models of human ocular diseases and perspective sections. LX wrote abstract and Morpholino-mediated knockdown approach in studying ocular diseases in zebrafish sections. Y-YZ wrote introduction section. Z-BJ constructed and revised the full minireview.

### Conflict of interest statement

The authors declare that the research was conducted in the absence of any commercial or financial relationships that could be construed as a potential conflict of interest.
